# Comparing Women's Contraceptive Preferences With Their Choices in 5 Urban Family Planning Clinics in Ghana

**DOI:** 10.9745/GHSP-D-16-00281

**Published:** 2017-02-08

**Authors:** Sarah D Rominski, Emmanuel SK Morhe, Ernest Maya, Abukar Manu, Vanessa K Dalton

**Affiliations:** a University of Michigan, Ann Arbor, MI, USA.; b Kwame Nkrumah University of Science and Technology, Kumasi, Ghana.; c University of Ghana, Accra, Ghana.

## Abstract

**Background:**

Concern about contraceptive side effects is a common reason reported by women for not using contraception or discontinuing use. We sought to characterize women's preferences related to method characteristics and side effects and to examine whether their adopted method was consistent with their stated preferences.

**Methods:**

Between June 1, 2015, and August 31, 2015, we surveyed women attending 5 urban family planning clinics in Kumasi and Accra, Ghana, before and after their counseling sessions. All women attending these clinics were approached to gauge their interest and eligibility for inclusion. Before counseling, women were asked about desired method characteristics and bothersome and intolerable side effects. After counseling, women were asked about method adoption and the counseling received about side effects. We then used crosstabs to compare the side effects women were counseled to expect, as well as those they reported would be intolerable, with their adopted methods to determine consistency between women's preferences and choices.

**Results:**

In total, 414 and 411 women completed the pre- and post-counseling surveys, respectively. The analysis sample consisted of 336 participants who adopted a method and were matched between the 2 surveys. The 3 most commonly chosen methods were the implant (n=135, 40.1%), injectables (n=109, 32.4%), and the intrauterine device (IUD) (n=52, 13.4%). The large majority (at least 87%) of method adopters chose a method that was well matched with their desired duration of effectiveness. Consistency between women's expressed intolerable side effects and their chosen methods was substantially lower: at least 70% of women choosing the implant, IUD, or injectables had stated they would stop using a method if they experienced those side effects that are in fact common with their respectively chosen methods. While 65.0% of those who adopted a method reported they were counseled to expect side effects, substantially less were counseled to expect the side effects common with use of their adopted method.

**Conclusion:**

Women's choice of contraceptive methods generally matched their stated preferences related to desired duration of effectiveness but not to potential side effects, and most women reported they were not counseled to expect the side effects common with use of their chosen method. Providers need to address potential side effects during counseling both to ensure women choose methods that will be a good fit with their desires and to reassure them that commonly experienced side effects are not harmful.

## Abstract (precis)

Women's method choice largely matched their stated desired duration of effectiveness but not their desires to avoid certain side effects. While most women reported they were counseled about side effects, many fewer reported being specifically counseled about common menstrual side effects with their chosen method, including side effects the women said would cause them to stop using the method.

## INTRODUCTION

Although use of effective contraception is growing in many parts of the world, only about 17% of women of reproductive age in sub-Saharan Africa use a modern contraceptive.^1^ Low levels of usage, however, do not indicate a lack of interest in family planning among women and their male partners; while fertility desires in many countries in sub-Saharan Africa are high, so too is the demand to both space and limit births, even among young women.^2^

Contraceptive use, as measured by the contraceptive prevalence rate (CPR), in Ghana has not changed significantly since a rapid increase from 12.9% in 1988 to 25.2% in 2003.^3^ The most recent Ghana Demographic and Health Survey showed a CPR in 2014 of 26.7%. Although the National Population Policy has an explicit objective “to ensure accessibility to, and affordability of, family planning means and services for all couples and individuals to enable them regulate their fertility,”^4^ the total fertility rate (TFR) in 2014 was 4.2 births per woman, a slight increase from the 2008 figure of 4.0 births per woman.^5^ This is in contrast to the marked decline in the fertility rate observed between the mid-1980s and the 1990s.

Unmet need for contraception is the percentage of women of reproductive age who want to stop or postpone childbearing but who report that they are not using a method to prevent pregnancy,^6^ and is used as an indicator of the gap between the demand for contraceptives and contraceptive use. Current unmet need for contraception is high in Ghana at about 30% of married women and 42% of sexually active unmarried women.^5^ This suggests potential high interest and demand for family planning among women and their partners.

Encouraging new adopters of contraceptive methods is important, but so too is understanding consistency of use among existing users.^7^ In 14 of 15 low-income countries, the majority of unplanned and unwanted births were the result of either contraceptive failure or discontinuation of a method for reasons other than a desire to get pregnant.^8^ Women's expectations of and experiences with side effects may lead to satisfaction with their method and continuation with using it or dissatisfaction and discontinuation of their method.^9^ Further, recent evidence suggests that the experience of side effects is increasingly a reason for why women have an unmet need for contraception.^10^ In Ghana specifically, intolerance of side effects is often an underlying reason for method discontinuation^11^^,^^12^ and is cited as the reason many women avoid initiating contraception.^13^ In recent years, health concerns and the experience of or concerns about side effects are increasingly driving non-use of contraception by Ghanaian women; the percentage of women who mentioned these as their reason for non-use increased from 14% in 1988 to 43% in 2008.^14^ This could, in part, be due to the heavy reliance on oral contraceptives and injectables,^5^ methods for which women report high levels of displeasure with side effects.

While there is a large body of evidence based on clinical trials detailing the possible physical symptoms and side effects that may be caused by using a particular contraceptive method, ranging from bleeding changes, headaches, breast tenderness, and weight change with hormonal methods to increased bleeding with the copper IUD,^15^^–^^17^ how users feel about these side effects is inherently subjective. In many settings, including Ghana, side effects and health concerns or fears are conflated.^13^ Further, Ghanaian women's unfavorable attitudes toward contraceptive methods, a key driver in use or non-use, originate from fears regarding the safety of these methods and intolerance of menstrual side effects, rather than from social or moral objections.^14^ Menstrual disruption is often considered to be a clinically benign side effect and is therefore sometimes minimized or dismissed by health personnel.^18^ However, many women have a low tolerance for menstrual changes, and thus side effects, and in particular bleeding changes, are a key reason for discontinuation of contraceptive methods.^19^ Understanding individuals' expectations of and tolerance for side effects is an important part of counseling and a means to ensuring satisfaction with the chosen method, and ultimately to ensuring consistent use of contraceptive methods.

Bleeding changes are a key reason for discontinuation of contraceptive methods.

In this study of Ghanaian family planning adopters, we aimed to (1) describe method characteristics women find desirable and side effects women report would be untenable; (2) describe the side effects women were counseled to expect from the method they adopted; and (3) characterize to what extent women chose methods that matched their desires for acceptable vs. intolerable side effects and other method characteristics.

This study aimed to characterize the extent women chose methods that matched their preferences for desired characteristics including side effects.

## METHODS

### Setting and Participant Recruitment

We conducted this cross-sectional study in urban family planning clinics of 2 teaching hospitals and 3 district hospitals in Kumasi and Accra, Ghana. Data were collected between June 1, 2015, and August 31, 2015. All women attending these clinics for family planning counseling and method choice were approached by a member of the study team and informed about the study to gauge their interest and eligibility for inclusion. Inclusion criteria consisted of being over the age of 18, intending to adopt a new method of family planning, and being able to converse in either English or Twi or Ga (the local languages). If the women met these inclusion criteria and agreed to participate, they were taken through a comprehensive verbal consent process. Consenting participants were interviewed both before and after their family planning counseling. This study used a convenience sample; all women who attended the clinic for family planning during the study period were invited to participate. No sample size calculations were conducted prior to study initiation.

### Survey Instrument

The survey was developed by the authors, an international study team with experience in family planning in Ghana. Questions were developed based on literature and expert opinion. The questionnaire was pretested for clarity and flow among women in the study clinics who met the inclusion criteria before the beginning of data collection. Revisions to the questionnaire were made based on this pilot testing. Questionnaire items included previous use of contraceptive methods and reasons for discontinuation, as well as what method of contraception women wanted to adopt, what method characteristics they desired, which side effects they would find bothersome, and which would be intolerable, causing them to discontinue the use of a contraceptive. After their counseling session, women were asked whether they were leaving with a method and, if they were, whether they were counseled to expect any side effects. If they had been counseled about side effects, they were asked what side effects they were counseled to expect. These answers were collected as free response and grouped. Multiple responses were maintained.

### Survey Administration

Interviews were conducted both before and after women's family planning counseling session in a private room near the family planning clinics. Only the woman and the research assistant were in the room where the interview took place, and no identifying information was collected. Phone numbers were used to link the pre- and post-counseling surveys. All data were collected on a Google tablet computer using Qualtrics software and results could not be seen once the form was completed. Questionnaires were interview-administered by trained research assistants.

### Data Analysis

To determine women's preferences, participants were asked 3 sets of questions. First, women were asked a variety of questions about method characteristics, such as, “I would prefer a method that I take every day.” The answers were recorded on a 5-point Likert scale from strongly disagree to strongly agree. We then created a dichotomous variable to represent yes/no agreement to the statement by grouping “strongly agree” and “agree” to indicate agreement with the statement, and “neither agree nor disagree,” “strongly disagree,” and “disagree” to indicate disagreement.

In the second set of questions to determine preferences, questions about side effects, such as, “I would not like a method if it stopped me from bleeding,” were asked also on a 5-point Likert scale. Similarly, “strongly agree” and “agree” were grouped, as were “neither agree nor disagree,” “strongly disagree,” and “disagree.”

Finally, participants were asked whether the experience of a variety of side effects would be intolerable enough to cause them to stop using the method. The answers to these questions were collected as a dichotomous variable (yes/no) and included such statements as, “Increased bleeding would cause me to stop using the method.”

In the post-counseling survey, participants were asked about the method they had adopted and also if they were counseled to expect side effects. Those who answered they were counseled to expect side effects were asked which side effects they were expecting. The side effects they were expecting, as well as those they reported as being so intolerable as to cause them to stop using the method, were compared with their adopted methods. Those who adopted methods that are known to cause such side effects were determined to have adopted a method that was not concordant with their preferences. For example, if a woman indicated decreased bleeding would be bothersome enough to cause her to stop using her method, but she adopted the injectable or the implant, she was determined to have adopted a method discordant with her preference. Women who reported they were counseled to expect side effects that are in fact shown in the literature to be caused by the method they adopted were determined to have been counseled appropriately to expect side effects common with the chosen method. For example, a woman who adopted an IUD and reported she was counseled to expect increased bleeding was determined to have been counseled to expect a side effect common with her chosen method. These comparisons were done using crosstabs in SPSS (Chicago, IL) version 22. Data are presented as descriptive statistics.

### Ethical Review

All study materials and methods were reviewed and approved by the Ghana Health Service Ethical Review Committee and the University of Michigan Institutional Review Board.

## RESULTS

### Background Characteristics

A total of 414 women completed the pre-counseling survey, and 411 completed the post-counseling survey. Of the original 414 participants, 336 left with a method and were matched between the 2 surveys (183 in Kumasi and 153 in Accra). A total of 55 participants did not leave with a method and an additional 23 participants from the pre-counseling survey could not be matched with the post-counseling survey. Thus, our analytical sample consisted of the 336 participants who had complete records.

Participants were generally well distributed across sociodemographic variables; the mean age of the total sample of 414 women who completed the pre-counseling survey was 29.3 years (range, 18 to 51; standard deviation, 6.7), and 248 participants (59.9%) were married (Table 1). Of the 411 women who completed the post-counseling survey, 337 (82.0%) left their counseling appointment with a method (1 participant who left with a method was not matched to the pre-counseling survey and thus was not included in the analytical sample). The primary reasons women gave for not leaving with a method were needing to wait for a pregnancy test (n=17), the clinic not having the supplies/equipment/providers necessary (n=13), having high blood pressure (n=9), and needing to consult with the husband (n=5).

**TABLE 1.** Background Characteristics of Participants, Kumasi and Accra, Ghana (N=414)

	Value
Age, years, mean (SD)	29.3 (6.7)
Married	248 (59.9)
Highest education	
None	31 (7.5)
Primary	71 (17.1)
Junior secondary	146 (35.3)
Senior secondary	89 (21.5)
More than secondary	77 (18.6)
Previously used a method(s)^a^	200 (48.3)
Pill	53 (26.5)
IUD	12 (6.0)
Injectable	97 (48.5)
Implant	36 (18.0)
Male condom	20 (10.0)
Female condom	3 (1.5)

Abbreviations: IUD, intrauterine device; SD, standard deviation.

Data presented as “number (%)” unless otherwise specified.

a Respondents could select more than 1 method.

Almost half of the sample (n=200, 48.3%) had previously used some form of contraception, with the injectable being the most commonly used method (n=97, 48.5%), followed by the pill (n=53, 26.5%) (Table 1).

### Women's Stated Preferences

**FIGURE 1:**
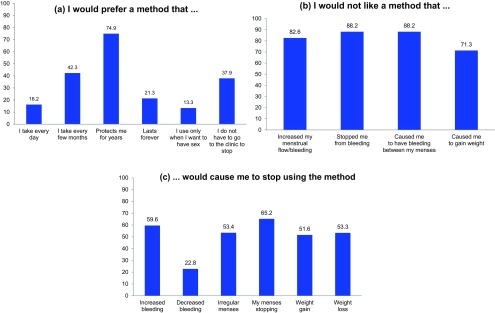
Women's Stated Preferences About Contraceptive Method Characteristics and Side Effects, Kumasi and Accra, Ghana (N=414) (percentage who agreed or strongly agreed with the statements)

The majority (n=310, 74.9%) of the full sample of participants agreed or strongly agreed that they would prefer a method that protects them for years (Figure 1a). A substantial number of women agreed or strongly agreed that they would prefer a method that they take every few months (n=175, 42.3%) or that lasts forever (n=88, 21.3%). Only 67 (16.2%) women agreed or strongly agreed they would prefer a method that they had to take every day. A majority (n=252, 60.9%) was not opposed to using a method that requires a visit to the facility to stop using it.

Participants had generally unfavorable attitudes toward bleeding changes caused by contraceptives. For example, a majority reported that increased bleeding (n=246, 59.4%), irregular bleeding (n=219, 52.9%), and amenorrhea (n=268, 64.7%) would be intolerable enough for them to stop using the method (Figure 1c). Decreased bleeding seemed to be more tolerable to women as only 94 women (22.8%) reported it would be intolerable enough to stop using the method.

### Women's Method Choices

The majority (n=187, 55.7%) of women who left with a method and were matched with the pre-counseling survey chose a long-acting reversible contraceptive (LARC); 52 women (15.5%) chose the IUD and 135 women chose the implant (40.2%) (Table 2). A substantial number (n=109, 32.4%) chose injectables. The remaining chose either sterilization (n=11, 3.3%) or short-acting methods (n=29, 8.6%).

**TABLE 2.** Women's Contraceptive Method Choices, Pre- and Post-Counseling, Kumasi and Accra, Ghana

Method	Preferred Method at Pre-Counseling (N=414)	Method Choice Post-Counseling (N=336)
Implant	172 (41.5)	135 (40.2)
Injectable	125 (30.2)	109 (32.4)
IUD	58 (14.0)	52 (15.5)
Pill	20 (4.8)	27 (8.0)
Female sterilization	17 (4.1)	11 (3.3)
Male condom	2 (0.5)	2 (0.6)
Don't know	20 (4.8)	NA

Abbreviation: IUD, intrauterine device.

All data are presented as “number (%).”

Most women in this study chose a long-acting reversible contraceptive.

### Counseling About Side Effects

Of the 336 participants who adopted a method of contraception, 218 (64.9%) reported they were counseled to expect side effects. Table 3 presents data on the percentage of women who reported being counseled to expect certain side effects by the method adopted; the side effects presented in the table are those side effects that are known to commonly occur with the method. Among the 52 women who adopted the IUD, only 16 (30.8%) reported they were counseled to expect an increase in bleeding (a commonly expected side effect with copper IUD use). Among the implant adopters, 28 (20.7%) reported they were counseled to expect irregular bleeding, while 14 (10.4%) and 37 (27.4%) reported they were counseled to expect decreased bleeding and no menses, respectively. For those using injectables, 46 (42.2%) reported they were counseled to expect their menses to stop.

Most women reported they were counseled to expect side effects, but much fewer reported being counseled to expect the side effects that commonly occur with their chosen method.

**TABLE 3.** Percentage of Women Who Reported Being Counseled to Expect Commonly Occurring Side Effects by Method Adopted

Method and Side Effect Counseled to Expect	No. (%)
Implant (N=135)	
Irregular bleeding	28 (20.7)
Decreased bleeding	14 (10.4)
No menses	37 (27.4)
Not counseled on any of these side effects	39 (28.9)
Injectables (N=109)	
Decreased bleeding	12 (11.0)
No menses	46 (42.2)
Weight gain	13 (11.9)
Not counseled on any of these side effects	34 (31.2)
IUD (N=52)	
Irregular bleeding	6 (11.5)
Increased bleeding	16 (30.8)
Not counseled on any of these side effects	26 (50.0)

Abbreviation: IUD, intrauterine device.

### Consistency Between Women's Method Choice and Stated Preferences

**FIGURE 2:**
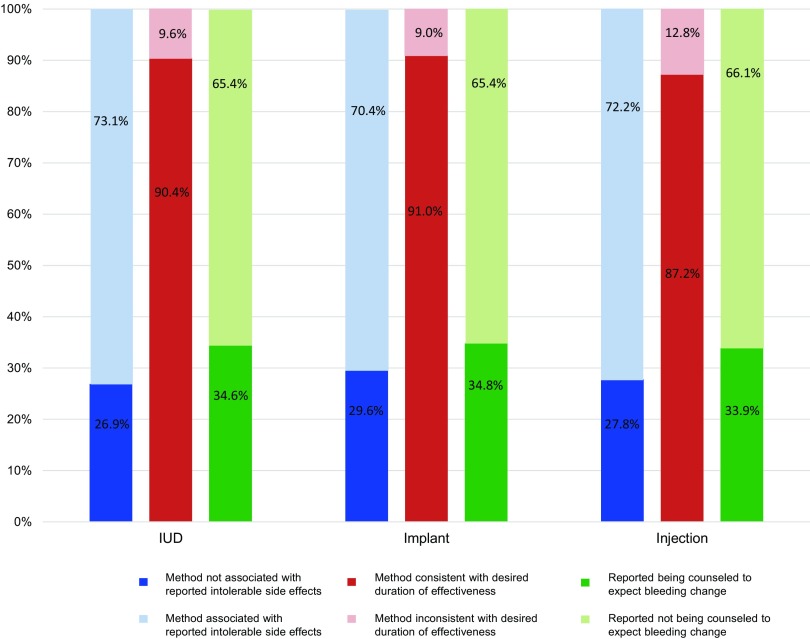
Consistency Between Women's Chosen Contraceptive Method and Their Stated Preferences for Duration of Effectiveness and Intolerable Side Effects, Along With Percentage of Women Counseled to Expect Bleeding Changes With Their Chosen Method

Figure 2 shows the concordance between women's method choice (for the 3 most popular methods—the IUD, the implant, and injectables) and their stated preferences at the pre-counseling survey with regard to desired duration of effectiveness and intolerable side effects (side effects that, if experienced, women stated they would stop using the method). The intolerable side effects included in the figure are those bleeding side effects that are commonly expected with use of that particular method; for example, increased bleeding for IUDs. Figure 2 also displays the proportion of method adopters who reported being counseled to expect bleeding changes with their chosen method and the proportion reporting they were not counseled to expect bleeding changes.

Among IUD adopters, 73.1% stated in their pre-counseling survey that experiencing increased bleeding would cause them to stop using the method, suggesting discordance with their chosen method because copper IUDs have been shown to increase bleeding among many users. Similarly, 70.4% of implant adopters stated at the pre-counseling survey that irregular, decreased, or no bleeding would cause them to stop using the method and 72.2% of injectable users stated that decreased or no bleeding would cause them to stop using the method, suggesting discordance since these are commonly experienced side effects with use of these methods. The majority (around 65%) of IUD, implant, and injectable adopters reported they were not counseled to expect bleeding changes. Bleeding changes are in fact expected to occur with all 3 of these methods.

73% of IUD adopters stated in their pre-counseling survey that experiencing increased bleeding would cause them to stop using the method.

There seemed to be greater concordance between women's choice of method and their stated desired duration of effectiveness: at least 87% of the adopters indicated a desired duration of effectiveness that lined up with the duration of effectiveness of their chosen method (a few months for injectables and years for LARCs).

The large majority of women chose methods that lined up with their stated preferences for duration of effectiveness.

## DISCUSSION

This study, conducted in urban areas of Accra and Kumasi, Ghana, sought to characterize women's contraceptive preferences and to examine whether their adopted method was consistent with their stated preferences. We found that the vast majority of women attending family planning clinics had a method in mind before the family planning counseling session, and many had strong preferences for and against method-specific qualities or side effects. It seems that most women received the method that they had been planning to use, and most women adopted a method that appeared consistent with some of their stated preferences, most notably duration of effectiveness. However, the majority of women left the clinics with a method that is known to cause the side effects they had characterized as intolerable—ones that would cause them to stop using the method. Furthermore, most women reported they were not counseled to expect these commonly occurring side effects.

Improving access to and use of modern contraception is an important strategy to both reduce maternal mortality and assist women to meet their fertility goals. Side effects are a key reason both for not using contraception and for discontinuing use of contraceptives.^10^^,^^14^ Women are diverse in preferences and providers need to guide them to the method that best suits their preferences. Our results show that while there is generally a good match with women's preferences regarding desired duration of effectiveness, many women are leaving with a method well known to cause a side effect they characterized as intolerable. It is not clear from these data if women were well informed about potential side effects; many did not report being educated on these side effects. Adopting a patient-centered model of care has been demonstrated to increase consistent contraceptive method use in high-resource settings,^20^ and has been tested on a limited basis in low-resource settings.^21^ More specifically, providers need to tailor information for women so that they know what side effects are common and not harmful and what things might cause temporary or longer-term discomfort, compared with potential (rare) complications to look for.

Many women in this study left with a method well known to cause a side effect they characterized as intolerable.

It appears that not all women in our study were provided with clear information about the side effects they should expect with the method they were given. Others too have reported that women are sometimes poorly informed about side effects,^2^ and this could lead to dissatisfaction and discontinuation. A recent analysis of the 2008 Ghana Demographic and Health Survey showed that 71% of IUD adopters discontinued the use of this method, mainly due to side effects and health concerns.^22^ Discussion of method side effects appears to not have changed much over the past decades. In 1995, Bongaarts and Bruce^23^ found that fewer than 50% of new adopters were counseled about their method's side effects and about 35% of women had discussions about how to manage these side effects should they arise.^23^ More recently, in Niger in 2012, only 40% of women reported being informed of possible side effects of methods.^24^ Some providers may be reluctant to discuss potential side effects for fear of the “nocebo” effect,^25^ the phenomenon where women experience side effects after being told to expect them because of the power of suggestion. However, evidence suggests that the discussion of negative side effects is not detrimental to method adoption and may be beneficial to method adherence.^26^^–^^28^ In fact, patients report that the reluctance of providers to discuss side effects makes them distrustful of counseling.^20^

Providers need training and support in good-quality counseling skills. The counseling session is an opportunity for providers to help align clients' desires and preferences with method characteristics so that clients receive a method that is likely to suit their needs, and thus one that they are more likely to continue using. Above all, it is the responsibility of providers to help women make an informed family planning choice by giving them information about the methods available to them, the characteristics of those methods, including side effects, and how to correctly use their chosen method. Given this information, some women may decide to sacrifice some of their preferences when those preferences do not align perfectly with available methods (for example, a woman who wants a long-acting method but finds menstrual changes bothersome may decide to use a long-acting method anyways because the other positive characteristics balance out the bleeding side effects). While supply-side barriers have long been determined to be factors limiting contraceptive uptake and continuation, demand-side barriers are increasingly being determined to be important as well.^14^ Once a woman overcomes the many potential barriers to adopting a method, the provider can help ensure her satisfaction with the chosen method by reassuring her that side effects are common with use and not harmful, which may improve her adherence to and continued use of the method. If women do not have good family planning experiences, this may erode their trust of the health care provider or health center and influence members of their social network to avoid using contraceptive methods. Clearly, more research is needed to fully understand the patient-provider interaction in this particular study setting.

The counseling session is an opportunity for providers to help align clients' desires and preferences with method characteristics.

Beyond the individual patient-provider relationship, it is possible that mass media campaigns aimed at reducing misinformation and myths about common side effects could help women to be more tolerant of innocuous side effects. The ability of women to use family planning is, of course, a larger societal issue, and many societal and cultural barriers reduce demand for family planning.^29^

The most frequently cited side effect that women said would cause them to stop using the method was amenorrhea, followed by increased bleeding. This finding supports research conducted in the 1970s, which suggested that amenorrhea was unacceptable to many women,^30^ although no African women were included in that study. In a qualitative study of women from a range of countries from the 1990s, women were “dismayed” by the possibility of a contraceptive method causing amenorrhea.^31^ However, in more recent work, Glasier and colleagues^32^ found that Nigerian women were generally willing to accept a method of contraception that induces amenorrhea, even though a majority said monthly bleeding was important to them. This could be indicative of a shift in the acceptability of a cessation in menses, although most women in our sample were not eager to adopt a method with such an effect. Education on the non-contraceptive health benefits of reduced blood loss associated with hormonal contraceptives, and reassurance that current global evidence-based guidance advises this is not harmful,^15^^,^^33^ could make some clients adopt methods with such a side effect.

### Limitations

This study is not without limitations. The relatively small sample size, as well as the fact that all study sites were located in urban areas, reduces the ability of these findings to be generalized. Further, these women were all seeking services at family planning clinics at district hospitals or tertiary care centers and thus might have been more interested in LARCs than women who seek contraceptive services from other locations. Qualitative investigation is important to develop clearer and deeper understanding of the concerns women have about contraceptives and what they expect from service providers. It is possible that counseling from the provider during the session changed women's perceptions toward side effects, and so their stated intolerance of a side effect before their visit changed post-counseling. These data would not capture a woman's change in preferences due to counseling. Finally, this cross-sectional survey does not allow us to determine to what extent women's reported desires matched actual behaviors. A follow-up, longitudinal study is important to assess if women's experience with either expected or unexpected side effects impacts contraceptive adherence.

## CONCLUSION

This study interviewed women before and after their contraceptive counseling session to determine to what extent women were receiving methods that matched their stated desires. Women had clear preferences regarding several contraceptive characteristics, including duration of effectiveness and side effects. It is important for providers to understand women's individual preferences and needs and how these preferences may interact with each other to guide women to make informed family planning choices. While women generally adopted methods that matched their stated desires related to duration of effectiveness, the adopted methods were less well matched to women's preferences regarding potential menstrual side effects. Future work could investigate whether the experience of side effects, both expected and unexpected, is associated with contraceptive adherence and continuation.

## Notes

**Peer Reviewed**

**First Published Online:** 2017 Feb 8
